# Ultrasonographic estimation of central venous pressure using the caudal vena cava to aorta ratio in dogs: a proof-of-concept study

**DOI:** 10.3389/fvets.2026.1807298

**Published:** 2026-05-11

**Authors:** Maxime Cambournac, Isabelle Goy-Thollot, Céline Pouzot-Nevoret

**Affiliations:** 1 Emergency and Critical Care Unit, Centre Hospitalier Vétérinaire Fregis, Paris, France; 2 Intensive Care Unit (SIAMU), VetAgro Sup, APCSe, Campus Vétérinaire de Lyon, Université de Lyon, Marcy l'Etoile, France

**Keywords:** canine, central venous pressure, hemodynamic monitoring, ultrasound, volaemia

## Abstract

**Context:**

Central venous pressure (CVP) might be used to guide fluid therapy in critically ill patients, but its invasive nature and associated risks have prompted growing interest in non-invasive alternatives such as ultrasonographic vascular indices.

**Objectives:**

To evaluate the correlation between the caudal vena cava (CVC) to aorta (Ao) (CVC:Ao) ratio and central venous pressure (CVP) and explore the use of this correlation for discriminating CVP values.

**Materials and methods:**

Intermittent CVP measurements were obtained using a water-filled fluid column in dogs admitted for renal replacement therapy. Low (LCVP), normal (NCVP) and high CVP (HCVP) were defined as values of <0, between 0 and 5, and >5 cmH_2_O (centimeters of water pressure), respectively. The sonographic assessment of volaemia (SAV) protocol was used to obtain the CVC:Ao ratio in the end-expiratory phase.

**Results:**

Fourteen dogs and 28 CVC:Ao ratio-CVP paired measurements were included. The CVP (in cmH_2_O) was estimated from the CVC:Ao ratio using the generated equation CVP = 20.9*(CVC:Ao)–19.44 (*R* = 0.69, *p* < 0.0001). The median CVC:Ao ratios differed significantly among groups at 0.91, 1.00, and 1.12 for LCVP, NCVP and HCVP, respectively (*p* = 0.0014). Using a CVC:Ao ratio <0.97, the sensitivity and specificity for detecting LCVP were 87.5 and 72.7%, respectively. Using a CVC:Ao ratio >1.09, the sensitivity and specificity for detecting HCVP were 66.7 and 90.9%, respectively.

**Clinical significance:**

The CVC:Ao ratio may be used as a surrogate non-invasive marker for estimating CVP in spontaneously breathing dogs. A CVP < 0 cmH_2_O is likely if the CVC:Ao ratio is <0.97, and a CVP > 5 cmH_2_O is likely if the ratio is >1.09.

## Introduction

Assessment of intravascular volume status is critical in managing critically ill and injured patients, particularly those requiring fluid resuscitation. Central venous pressure (CVP), defined as the pressure within the intrathoracic cranial or caudal vena cava relative to atmospheric pressure, has historically served as a surrogate for right atrial pressure and a clinical proxy for systemic preload (1). In both human and veterinary medicine, CVP has been widely used to evaluate circulating blood volume and to guide fluid therapy (2, 3).

Despite its widespread use, CVP monitoring presents several limitations. While both low and high CVP values have been associated with increased mortality across various clinical conditions, invasive catheterization remains necessary for measurement (4, 5). This approach requires additional technical resources and is associated with significant risks, including arrhythmias, vascular and cardiac injury, pneumothorax, thrombosis, infection, and phlebitic syndrome (6). For these reasons, interest in reliable, non-invasive surrogates of CVP has grown considerably.

Ultrasonography offers a fast, safe, and repeatable bedside tool to assess intravascular volume status. In human medicine, sonographic parameters such as inferior vena cava (IVC) diameter, collapsibility index (CI), and the IVC-to-aorta (IVC:Ao) ratio have been extensively investigated as non-invasive alternatives to invasive CVP measurement (7, 8). The IVC:Ao ratio in particular is considered attractive because it partially corrects for individual body size and exhibits relatively low interobserver variability (9, 10). However, the overall diagnostic accuracy and reproducibility of these indices remain a subject of debate.

Several studies have reported conflicting results regarding the strength of the correlation between sonographic IVC parameters and CVP. In a systematic review including over 1,400 patients, Ciozda et al. reported correlation coefficients between 0.5 and 0.7, highlighting significant heterogeneity among studies (7). For example, Parenti et al. found that IVC diameter correlated only moderately with CVP (*r* = 0.36), and jugular vein measurements were in fact more predictive (*r* = 0.58) (11). In neonates, Mugloo et al. reported excellent correlation between IVC-CI and CVP (*r* = −0.968), but these results are difficult to generalize due to anatomical and physiological differences (12). A recent meta-analysis by Chaves et al. including over 3,100 fluid challenge episodes, concluded that static markers like CVP had a lower predictive value for fluid responsiveness (AUC = 0.77) compared to dynamic indices such as ∆IVC (AUC = 0.83) or pulse pressure variation (13). These findings have led to recommendations against relying on CVP alone for fluid decisions, emphasizing instead the value of multimodal or dynamic assessment.

In veterinary medicine, evidence remains limited. Most studies evaluating CVC dimensions have been performed in anesthetized or experimentally ventilated dogs, with relatively few data available in conscious clinical patients (29, 30). The sonographic assessment of volaemia (SAV) protocol, validated for reproducibility in dogs, provides a standardized method to measure the CVC:Ao ratio (14). Recently, reference values for the CVC:Ao ratio in healthy adult dogs have been proposed, with reported ranges from 0.93 to 1.32, supporting its potential use as a baseline for clinical interpretation (15). Beyond healthy populations, this ratio has been shown to decrease in hypovolemic states such as hemorrhagic shock (15), and to increase in conditions associated with venous congestion, such as pericardial effusion (16), highlighting its utility across a spectrum of volume-related disorders. However, the correlation between this ratio and invasively measured CVP in spontaneously breathing dogs has not yet been established. Our study aims to evaluate the correlation between the CVC:Ao ratio and invasively measured CVP in spontaneously breathing dogs using a standardized ultrasonographic protocol.

We prospectively evaluated the correlation between CVC:Ao ratio measurements obtained via the SAV protocol and invasively measured CVP in spontaneously breathing dogs with acute kidney injury requiring renal replacement therapy. We also assessed the ability of the CVC:Ao ratio to differentiate low, normal, and high CVP states. We hypothesized that the CVC:Ao ratio would correlate positively with CVP and could serve as a reliable, non-invasive indicator of intravascular volume status in critically ill dogs.

## Materials and methods

This prospective study was approved by the institutional ethics committee of VetAgro Sup (Record Number: 1650). Dogs admitted to the intensive care unit of VetAgro Sup between May 2016 and July 2017 for acute kidney injury requiring at least two sessions of renal replacement therapy were enrolled. Dogs were eligible for inclusion if they met all of the following criteria: confirmed AKI diagnosis requiring at least two sessions of renal replacement therapy, availability of a functional central venous catheter allowing CVP measurement, and successful acquisition of a CVC:Ao ratio measurement prior to each dialysis session. Indications for initiating dialysis followed our standard criteria and were consistent with the general recommendations (17). These included a serum creatinine concentration >60 μmol/L, persistent oliguria or anuria, and refractory hyperkalemia despite medical management. Vascular access was obtained using 11.5 French, 15-cm double-lumen dialysis catheters, in accordance with clinical guidelines and as part of our standard dialysis management protocol. Catheter tip placement was confirmed radiographically. The catheter tip was aimed to be positioned in the right atrium, and correct placement was confirmed on thoracic radiography prior to each measurement session. Dogs were excluded if pericardial effusion was identified on thoracic POCUS, as this condition causes elevated CVP through impaired cardiac filling and would have confounded the correlation between the CVC:Ao ratio and CVP. Dogs were also excluded if either the CVP measurement or the CVC:Ao ratio could not be obtained.

### Ultrasound measurement of CVC:Ao ratio

All measurements were performed with the same device (X-Porte Vet, FUJIFILM Sonosite Inc.) using a 5–8-MHz convex transducer. The sonographic protocol was performed as previously described, also referred to as the sonographic assessment of volaemia (SAV) protocol (14). Briefly, alcohol was applied to the area over the left kidney. In right lateral recumbency, the left kidney was identified, and the probe was moved cranially and then oriented dorsally to visualize the caudal vena cava (CVC) and aorta (Ao) with the cranial part of the left kidney in the same field. The mean diameters of both vessels were measured immediately in B-mode at the end of the expiratory phase and used to calculate the CVC:Ao ratio. All ultrasonographic measurements were performed by the same operator (MC), who was blinded to the CVP value.

### Data record

The recorded data included signalment, CVC:Ao ratio, and CVP measurements. Central venous catheters were considered functional if blood could easily be aspirated and flushed. CVP was measured using a water-filled column manometer connected to the distal lumen of the central venous catheter. The system was flushed before each measurement, and the manometer was zeroed at the level of the sternum with the dog in right lateral recumbency. Central venous pressure measurements were performed by the same ICU specialized nurse using the following standardized procedure (18): after equilibration of the water column with the patient’s venous pressure, the height of the fluid column was recorded in cmH_2_O. Because heartbeat and respiration may cause minor fluctuations in the column, the CVP reading was taken visually at the bottom center of the floating meniscus, during the end-expiratory phase. For each dog, the CVC:Ao ratio and CVP were measured approximately 1 h before both the first and the second renal replacement therapy sessions. The CVC:Ao ratio was measured within 5 min after each CVP measurement. The inclusion of two measurement time points per animal was intentional: by capturing paired data before each of the two dialysis sessions, we aimed to sample a broader range of CVP values and to document potential intra-individual variation in both CVP and CVC:Ao ratio over time, thereby strengthening the dataset for correlation and diagnostic analyses. To evaluate the ability of this ratio to discriminate between CVP values, the dogs were divided into three groups: (1) dogs with a low CVP (LCVP) value, defined by CVP measurements <0 cmH_2_O; (2) dogs with a normal CVP (NCVP) value, defined by CVP measurements between 0 and 5 cmH_2_O; and (3) dogs with a high CVP (HCVP) value, defined by CVP measurements >5 cmH_2_O (1).

### Statistical analysis

No formal *a priori* sample size calculation was performed, as this study was intended as a preliminary proof-of-concept investigation. The sample size reflects the number of dogs meeting all inclusion criteria during the study period, a constraint inherent to studies involving rare critically ill populations requiring renal replacement therapy. As assessed for normality with a D’Agostino–Pearson omnibus test, certain variables were non-normally distributed. For ease and consistency, all data are presented as the median (range), and a 95% confidence interval (95% CI) may be reported if considered of interest. Linear regression was used to evaluate the correlation between the CVC:Ao ratio (predictor variable) and CVP (dependent variable). Although each dog contributed two paired measurements, simple linear regression was used as an exploratory analysis. The three dogs excluded due to catheter dysfunction prior to the second session were not included in the analysis, as they did not fulfill the predefined inclusion criteria requiring two complete paired measurement sessions. The potential lack of independence between repeated measurements is acknowledged as a limitation. Ideally, a linear mixed-effects model including individual dog as a random effect would offer more appropriate statistical treatment but was not performed due to the small sample size. The distribution of CVC:Ao ratio measurements across the LCVP, NCVP, and HCVP groups was compared using a Kruskal–Wallis test. Similarly, this analysis does not account for repeated measures, and the use of mixed models would be recommended for future studies with larger datasets. Receiver operating characteristic (ROC) curves and associated area under the curve (AUC) values were calculated to evaluate the ability of the CVC:Ao ratio to predict CVP values and discriminate between the three CVP categories. Cut-off values that maximized specificity and sensitivity were determined using the Youden index (19). A Bland–Altman analysis was performed to evaluate the agreement between CVP values estimated from the regression equation and invasively measured CVP values, both expressed in cmH_2_O. One measurement pair was identified as an outlier based on a difference exceeding 2.5 standard deviations from the mean (CVP estimated: +3.77 cmH_2_O; CVP measured: −5 cmH_2_O; difference: +8.77 cmH_2_O) and was excluded from this analysis. All other statistical analyses were performed on the complete dataset of 28 paired measurements. Statistical analyses were performed, and figures were generated using commercial software (GraphPad Prism 7, GraphPad Software Inc.). A *p*-value < 0.05 was considered statistically significant.

## Results

### Animals

Seventeen dogs met the initial inclusion criteria during the study period. Three dogs were subsequently excluded because CVP measurements could not be obtained prior to the second renal replacement therapy session due to an inability to flush the catheter, likely caused by suspected clotting. A total of fourteen dogs were therefore included in the final analysis, each contributing two measurement points—one before each of the first two dialysis sessions. The median age of included dogs was 4.4 years (range: 0.25–15 years), and the median body weight was 25.3 kg (range: 11.4–29.3 kg). Six dogs were purebred, including two Golden Retrievers and one each of American Staffordshire Terrier, Beauceron, Boxer, and Akita Inu. All dogs were diagnosed with leptospirosis-related acute kidney injury (AKI), based on consensus criteria (20).

### Correlation between CVC:Ao ratio and CVP

**Figure 1:**
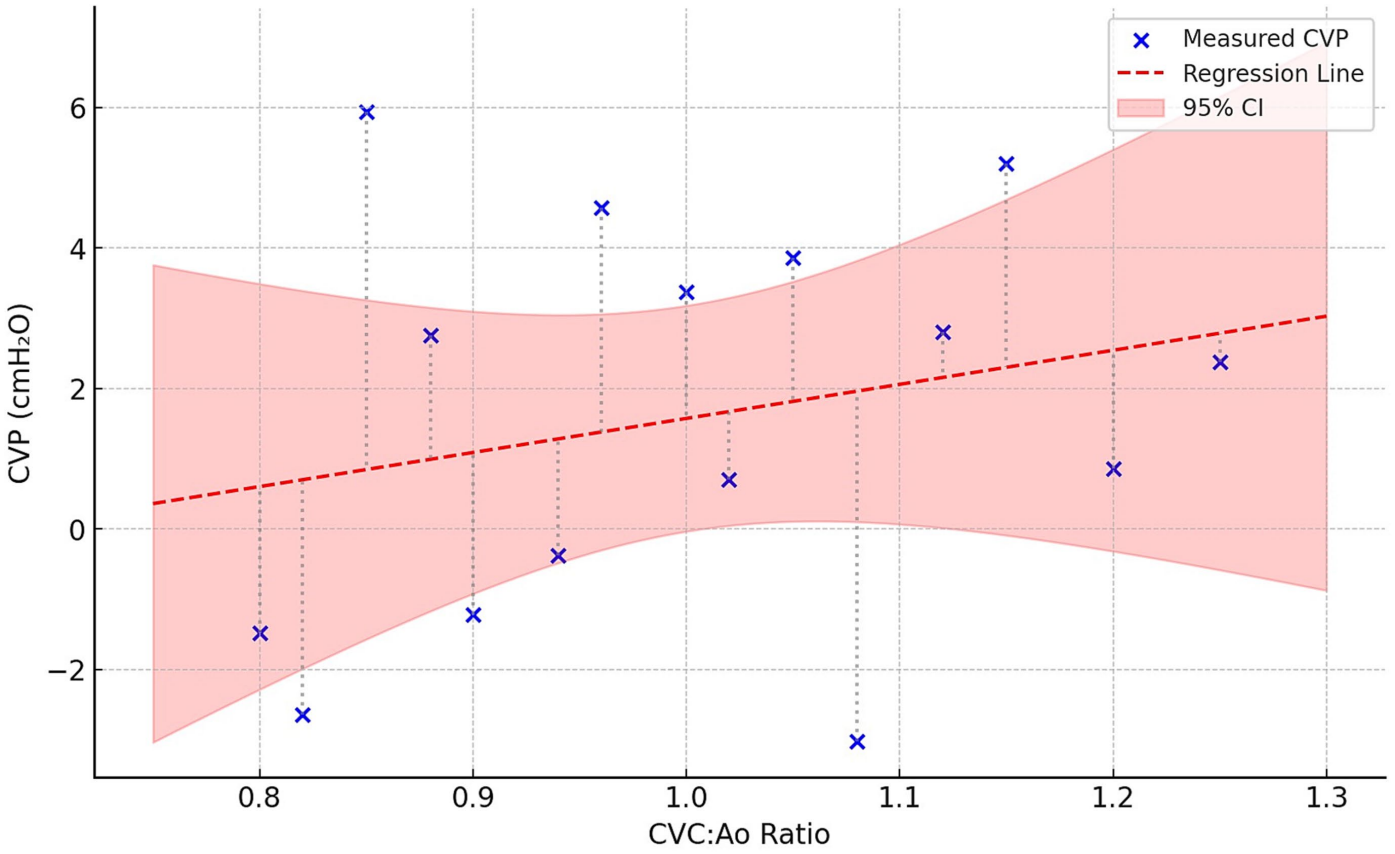
Linear regression between the CVC:Ao ratio and CVP values, with 95% confidence interval and residuals.

A total of 28 paired measurements of CVC:Ao ratio and CVP were obtained. Linear regression analysis revealed a statistically significant correlation between the CVC:Ao ratio and CVP (*R* = 0.69, *p* < 0.0001). The regression model generated the following equation: CVP (cmH2O) = 20.9 × (CVC:Ao) – 19.44. The regression model is represented in Figure 1. Visual inspection of the regression plot demonstrated a clear positive trend, with most values clustering around the regression line despite some physiological variability between individual dogs.

### CVP group differentiation based on CVC:Ao ratio

**Figure 2:**
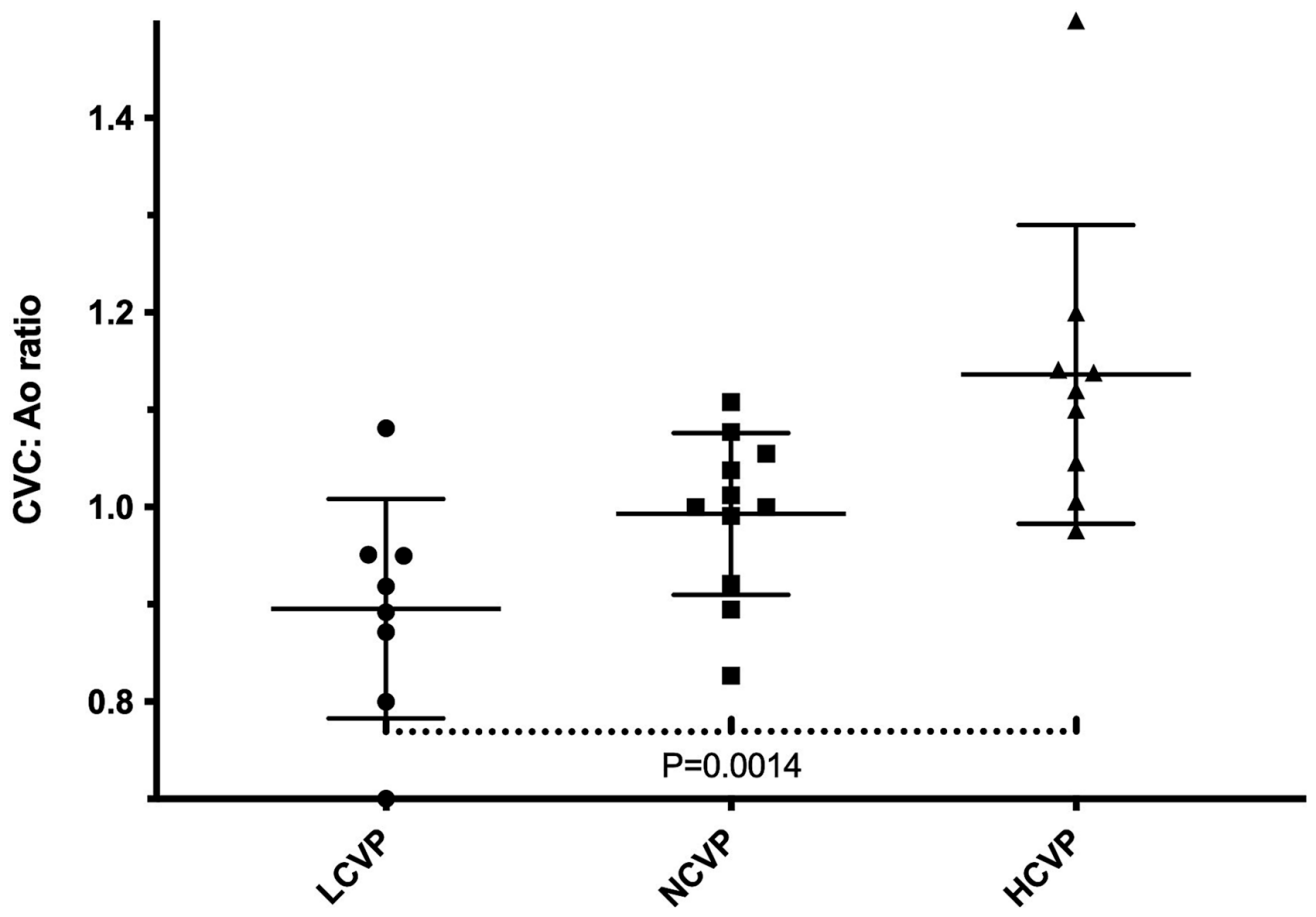
Box-and-whisker plot of CVC:Ao ratios according to CVP categories (LCVP, NCVP, HCVP).

The median CVP values in each group were −3 (range: −9 to −2), 3 (range: 1–5), and 6 (range: 6–8) cmH2O for the LCVP, NCVP, and HCVP groups, respectively. Corresponding median CVC:Ao ratios were 0.91 (95% CI: 0.80–0.90), 1.00 (95% CI: 0.94–1.05), and 1.12 (95% CI, 1.02–1.25), respectively. Table 1 summarize CVC:Ao ratio according to CVP groups, and Figure 2 represents box plot of CVC:Ao ratio variation relative to CVP group. A statistically significant difference in CVC:Ao ratios was observed among the three groups (*p* = 0.0014).

**Table 1:** Median (range) of CVP and median (95% CI) of CVC:Ao ratio in the 3 groups.

Group	Median CVP (cmHâ‚ O)	CVP range	Median CVC:Ao	95% CI
LCVP	−3	(−9, −2)	0.91	(0.8, 0.9)
NCVP	3	(1, 5)	1	(0.94, 1.05)
HCVP	6	(6, 8)	1.12	(1.02, 1.25)

### Diagnostic performance of the CVC:Ao ratio

**Figure 3:**
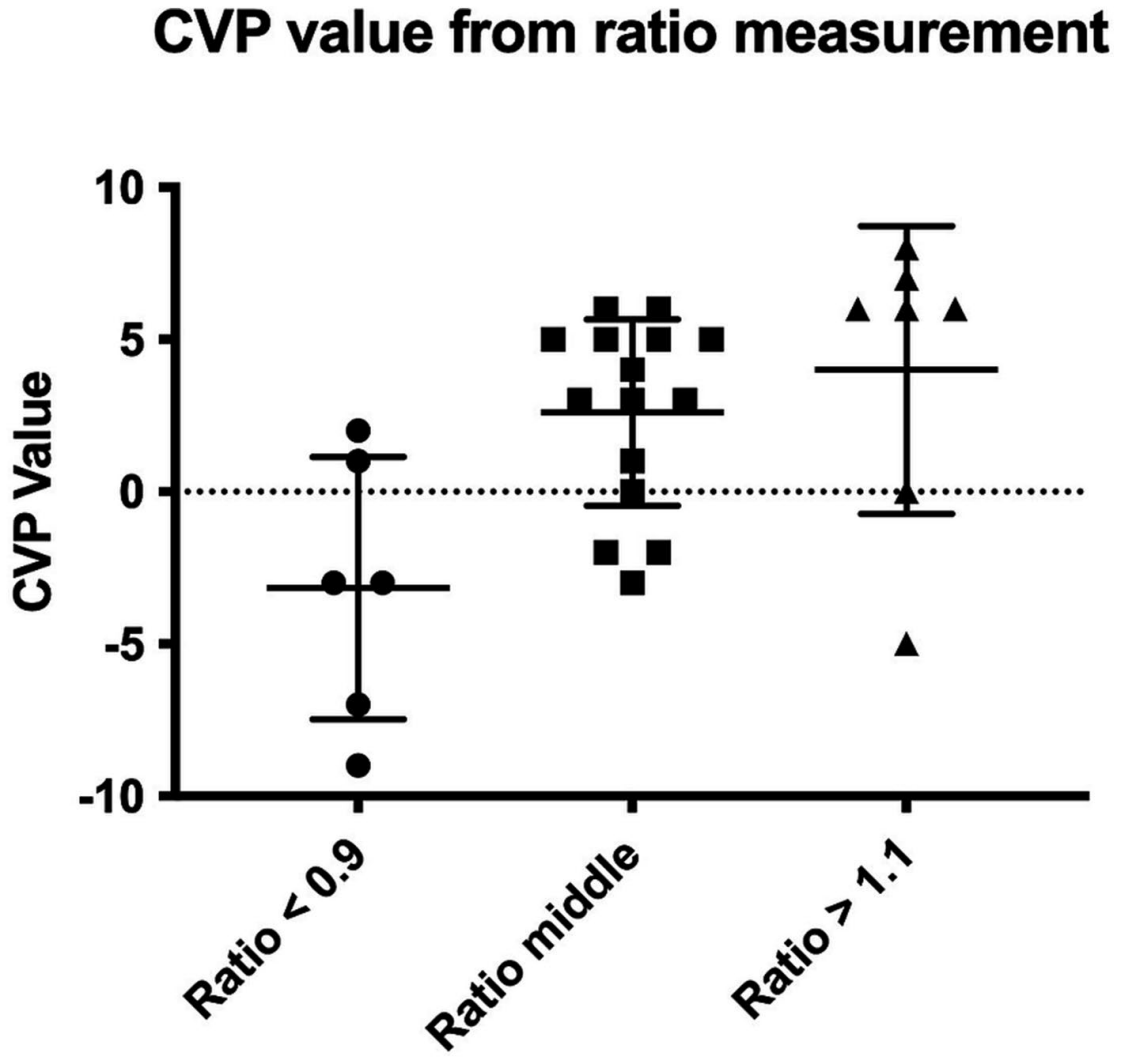
Distribution of individual central venous pressure (CVP) measurements according to the corresponding measured caudal vena cava to aorta (CVC:Ao) ratio.

**Figure 4:**
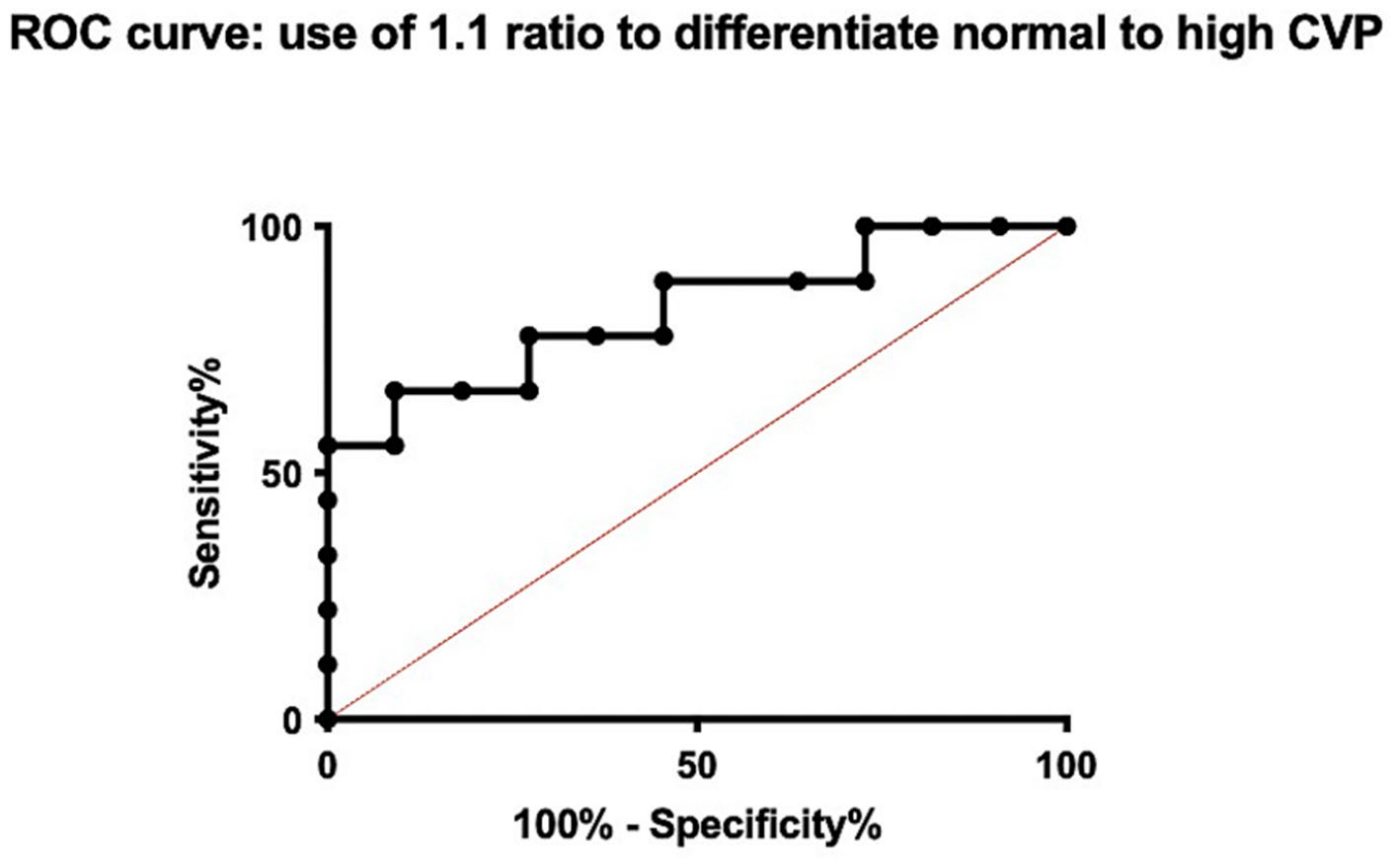
Receiver operating characteristic (ROC) curve for the ability of the caudal vena cava to aorta (CVC:Ao) ratio to predict low central venous pressure (LCVP) compared to normal CVP (NCVP).

**Figure 5:**
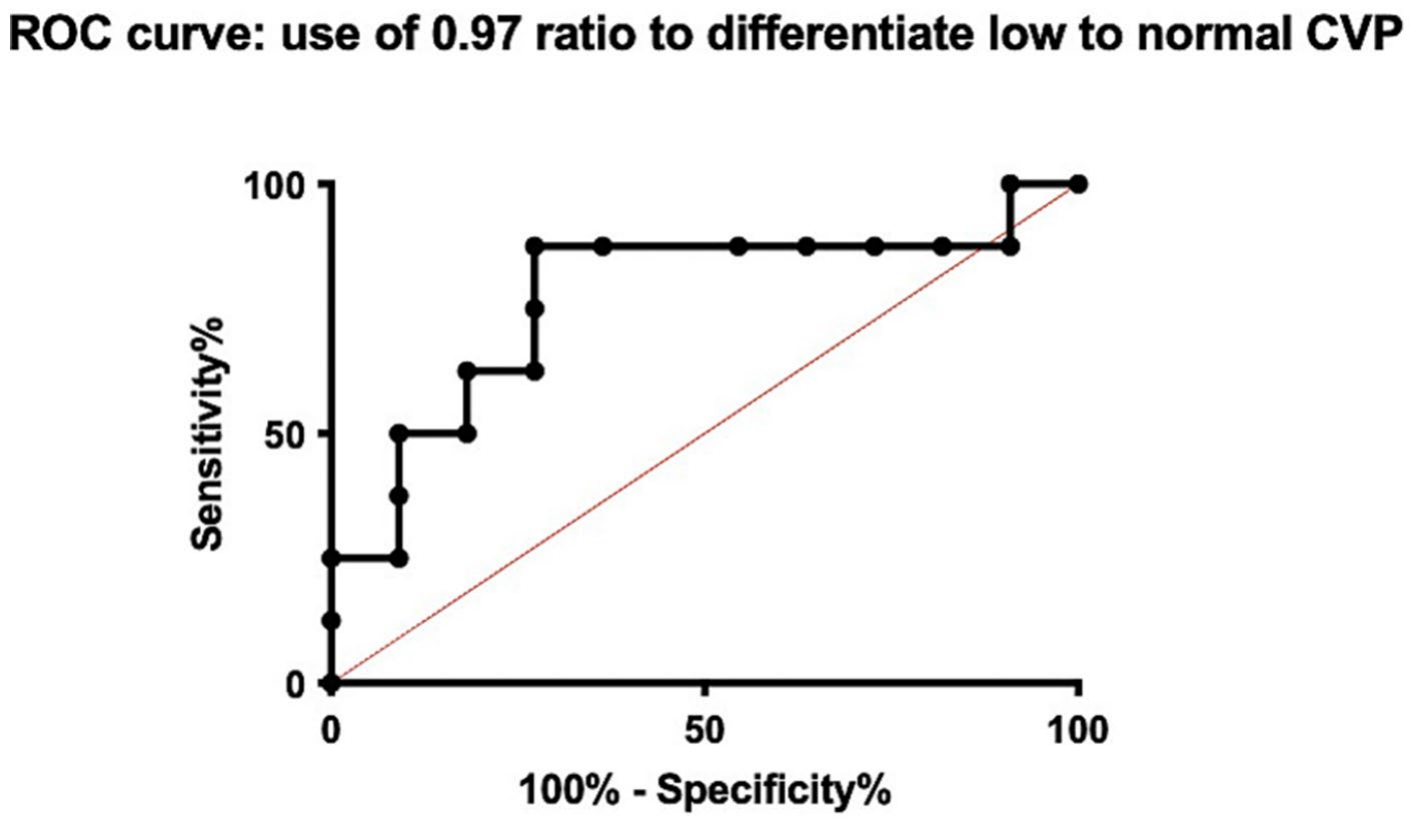
Receiver operating characteristic (ROC) curve for the ability of the caudal vena cava to aorta (CVC:Ao) ratio to predict high central venous pressure (HCVP) compared to normal CVP (NCVP).

To visualize the diagnostic performance of the CVC:Ao ratio, individual CVP measurements were plot against the measured CVC:Ao ratio, enabling assessment of the distribution and overlap of CVP values for each ratio category (Figure 3). Receiver operating characteristic (ROC) curve analysis was used to evaluate the performance of the CVC:Ao ratio in predicting low and high CVP states. To differentiate LCVP from NCVP, the cut-off of CVC:Ao ratio < 0.97 yielded an area under the curve (AUC) of 0.77 (Figure 4). At this threshold, the sensitivity for identifying low CVP was 87.5%, and the specificity was 72.7%. To differentiate HCVP from NCVP, the CVC:Ao ratio cut-off of > 1.12, was associated with AUC of 0.83 (Figure 5). The corresponding sensitivity and specificity were 66.7 and 90.9%, respectively.

### Bland–Altman analysis

**Figure 6:**
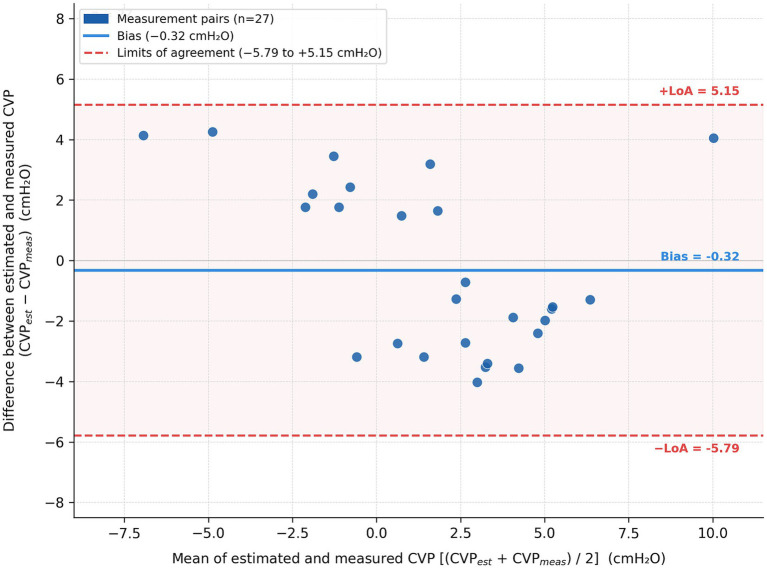
Bland–Altman plot comparing CVP values estimated from the regression equation with invasively measured CVP values (*n* = 27 paired measurements; one outlier excluded—see statistical analysis). The *x*-axis represents the mean of estimated and measured CVP values; the *y*-axis represents their difference (CVP estimated − CVP measured). The solid blue line indicates the mean bias (−0.32 cmH_2_O; 95% CI: −1.37 to +0.73). Dashed red lines indicate the limits of agreement (−5.79 to +5.15 cmH_2_O, corresponding to ±1.96 SD). No data points fell outside the limits of agreement.

One measurement pair was identified as an outlier (CVC:Ao ratio: 1.108; CVP measured: −5 cmH_2_O; CVP estimated: +3.77 cmH_2_O; difference: +8.77 cmH_2_O) and excluded from the Bland–Altman analysis as described in the Statistical Analysis section. Bland–Altman analysis of the remaining 27 paired measurements revealed a mean bias of −0.32 cmH_2_O (95% CI: −1.37 to +0.73). The limits of agreement ranged from −5.79 to +5.15 cmH_2_O, with no data points falling outside these limits. These results are illustrated in Figure 6.

## Discussion

In this prospective study, our results demonstrated a significant correlation between the CVC:Ao ratio measured using the SAV protocol and CVP in spontaneously breathing dogs with acute kidney injury requiring renal replacement therapy. The correlation coefficient of 0.69 between CVC:Ao and CVP was consistent with values reported in human studies (ranging from 0.39 to 0.89), confirming our results and the relevance of the CVC:Ao ratio to estimate CVP (7, 21, 22).

A key objective of this study was to evaluate the discrimination capacity of the CVC:Ao ratio associated with clinically relevant CVP ranges. The CVC:Ao ratio was significantly different among the three CVP groups. Dogs with low CVP (<0 cmH_2_O) had a median CVC:Ao of 0.91, those with normal CVP (0–5 cmH_2_O) had a median of 1.00, and those with high CVP (>5 cmH_2_O) had a median of 1.12.

In human medicine, the evaluation of the use of the CVC:Ao ratio as a surrogate of CVP has motivated many studies (5, 21, 23–25). Among these studies, mean IVC/Ao ratios ranging from 0.7 ± 0.09 SD in hypovolemic patients to 1.2 ± 0.12 SD in euvolemic patients and 1.6 ± 0.05 SD in volume-overloaded patients have been described. While those data are echoing our results, human studies are not always directly transposable to veterinary medicine. Similarly, in a recent study by Combet-Curt et al. (15), 29 dogs with spontaneous circulatory shock had a median CVC:Ao ratio of 0.82 (IQR: 0.76–0.87) before receiving fluid therapy, and 0.92 (IQR: 0.82–1.02) after resuscitation. Notably, 28 out of 29 dogs had a CVC:Ao ratio <0.93 at admission. These values are in agreement with the low ratios found in our LCVP group, highlighting the ability of the CVC:Ao ratio to reflect clinically relevant hypovolemia.

Conversely, our results confirm that a CVC:Ao ratio >1.09 is predictive of high CVP in spontaneously breathing dogs, potentially indicating a hypervolemic or congestive state. While fluid overload can be difficult to detect using conventional clinical or laboratory parameters, the CVC:Ao ratio may offer a rapid, repeatable, and non-invasive tool to support such assessments, as has been advocated in human critical care (26, 27). Interestingly, this finding is consistent with recent studies that reported significantly elevated CVC:Ao ratios in dogs with pericardial effusion and cardiac tamponade (16). In this study, the median CVC:Ao ratio in tamponade cases reached 1.15, a value comparable to the median ratio of 1.12 observed in our high CVP group. Cardiac tamponade is a cause of elevated CVP due to impaired cardiac filling and consequent venous congestion. The similarity between these values reinforces the concept that an elevated CVC:Ao ratio reflects increased right-sided venous pressure, regardless of the underlying etiology. These consistent findings across two distinct clinical contexts suggest that the CVC:Ao ratio may serve as a reliable indicator of venous congestion and volume overload in dogs.

Reported CVC:Ao ratios in 60 clinically healthy dogs ranged from 0.93 to 1.32, supporting the interpretation that values close to 1.0 reflect a normovolemic state, in veterinary medicine too (28). In human medicine, sonographic IVC/Ao for fluid status in young individuals from the American Journal of Emergency Medicine [18] concluded that for the healthy young population, the IVC/Ao reference value is 1.2 ± 0.17 SD. These findings, together with the current literature, strongly support a normal CVC:Ao ratio of approximately 1.0.

Based on the results of the present study, as well as the integration of the literature, the CVC:Ao ratio might serve to differentiated between low, normal, and high CVP states. However, the clinical applicability rely on sensitivity and specificity, as in a clinical setting, the ability to discriminate between CVP categories and limit misclassification is a cornerstone of any diagnostic tool. In our study, a CVC:Ao ratio <0.97 predicted low CVP with 87.5% sensitivity and 72.7% specificity, while a ratio >1.12 predicted high CVP with 66.7% sensitivity and 90.9% specificity. These findings are consistent with previously published data in humans where sensitivity and specificity range from 65 to 95. While such values are acceptable, the moderate sensitivity to differentiate CVP values has to be considered for clinical application, as this ratio might not detect all low CVP value. In opposite, the good specificity of this ratio validate the diagnostic capacity with a low false positive. In a clinical context, this is in accordance with previous study, as the CVP is the pressure applied, and not only the volume. This highlight also that a normal ratio might still be associated with either low, normal or high CVP values. From a clinical standpoint, the authors advocate that a ratio under or above the cutoff is in favor of low or high CVP, while a normal ratio should be interpreted with cautious, and in regard to the clinical context. Importantly, all measurements were obtained in spontaneously breathing dogs, without sedation or mechanical ventilation, which enhances the translational value of the findings to routine clinical settings.

Several limitations should be acknowledged. The relatively small number of dogs may limit statistical power. Although each dog contributed two measurement points, these repeated data points may not be entirely independent. Bland–Altman analysis demonstrated a near-zero mean bias (−0.32 cmH_2_O) between estimated and measured CVP values, confirming the absence of systematic error in the regression model. However, the limits of agreement (−5.79 to +5.15 cmH_2_O) were relatively wide, consistent with the moderate correlation coefficient (*R* = 0.69) and the inherent biological variability of a small proof-of-concept cohort. These findings support the interpretation that the CVC:Ao ratio should be regarded as a clinical screening tool rather than a precise substitute for invasive CVP measurement.

One measurement pair was excluded from the Bland–Altman analysis due to a discrepancy of +8.77 cmH_2_O (CVC:Ao ratio: 1.108; CVP measured: −5 cmH_2_O; CVP estimated: +3.77 cmH_2_O). This discrepancy was attributed to a likely artifact affecting the invasively measured CVP value, rather than a true physiological state, as a CVC:Ao ratio of 1.108 is not consistent with a markedly negative CVP. Several mechanisms may account for this measurement error. First, increased respiratory effort in a critically ill patient can generate marked inspiratory thoracic pressure swings, transiently lowering intrathoracic pressure and thus the water-column CVP reading, particularly if the measurement was not strictly captured at end-expiration. Second, partial obstruction of the catheter lumen may have dampened the transmission of venous pressure to the manometer, resulting in an artificially low reading. Third, despite the standardized protocol, inadvertent measurement outside the end-expiratory phase cannot be entirely excluded. Each of these artifacts would selectively lower the measured CVP without affecting the CVC:Ao ratio, thereby producing the observed discordance.

While this limitation was accounted for in the interpretation of the statistical analysis, future studies should consider linear mixed models to address repeated-measure designs. In addition, although catheter placement was radiographically verified, operator variability and anatomical differences among patients could have influenced CVP accuracy. Respiratory variation and pressure applied to the ultrasound probe can also affect CVC measurement, though steps were taken to standardize acquisition and minimize such effects, as all ultrasound measurements were obtained at end-expiration, and the anatomical positioning near the left kidney under the last rib helped reduce transducer compression. Additionally, the absence of simultaneous measurements of the caudal vena cava collapsibility index and the plethysmographic variability index (PVi) represents a limitation, as correlating these dynamic indices with the CVC:Ao ratio could have further informed its clinical applicability. Finally, missing measurements resulting from catheter dysfunction may have introduced a degree of selection bias.

In conclusion, the CVC:Ao ratio appeared to be well correlated with the CVP measurement. A CVC:Ao ratio < 0.97 is strongly indicative of a low CVP, while a ratio > 1.09 is indicative of a high CVP. Caution should be exercised when interpreting a normal CVC:Ao ratio.

## Data Availability

The original contributions presented in the study are included in the article/supplementary material, further inquiries can be directed to the corresponding author.
